# Impaired cholesterol metabolism in the mouse model of cystic fibrosis. A preliminary study

**DOI:** 10.1371/journal.pone.0245302

**Published:** 2021-01-07

**Authors:** Felice Amato, Alice Castaldo, Giuseppe Castaldo, Gustavo Cernera, Gaetano Corso, Eleonora Ferrari, Monica Gelzo, Romina Monzani, Valeria Rachela Villella, Valeria Raia

**Affiliations:** 1 Dipartimento di Medicina Molecolare e Biotecnologie Mediche, University of Naples Federico II, Naples, Italy; 2 CEINGE-Biotecnologie Avanzate, Scarl, Naples, Italy; 3 Dipartimento di Scienze Mediche Traslazionali, University of Naples Federico II, Naples, Italy; 4 Dipartimento di Medicina Clinica e Sperimentale, University of Foggia, Foggia, Italy; 5 European Institute for Research in Cystic Fibrosis, San Raffaele Scientific Institute, Milan, Italy; 6 Dipartimento di Scienze della Salute, University of Eastern Piedmont, Novara, Italy; National Research Council of Italy, ITALY

## Abstract

This study aims to investigate cholesterol metabolism in a mouse model with cystic fibrosis (CF) by the comparison of affected homozygous versus *wild type* (WT) mice. In particular, we evaluated the effects of a diet enriched with cholesterol in both mice groups in comparison with the normal diet. To this purpose, beyond serum and liver cholesterol, we analyzed serum phytosterols as indirect markers of intestinal absorption of cholesterol, liver lathosterol as indirect marker of *de novo* cholesterol synthesis, liver cholestanol (a catabolite of bile salts synthesis) and the liver mRNA levels of *LDL receptor* (*LDLR*), *3-hydroxy-3-methylglutaryl-CoA reductase* (*HMG-CoAR*), *acyl CoA*:*cholesterol acyl transferase 2* (*ACAT2*), *cytochrome P450 7A1* (*CYP7A1*) and *tumor necrosis factor alpha* (*TNFα*). CF mice showed lower intestinal absorption and higher liver synthesis of cholesterol than WT mice. In WT mice, the cholesterol supplementation inhibits the synthesis of liver cholesterol and enhances its catabolism, while in CF mice we did not observe a reduction of *LDLR* and *HMG-CoAR* expression (probably due to an altered feed-back), causing an increase of intracellular cholesterol. In addition, we observed a further increase (5-fold) in *TNFα* mRNA levels. This preliminary study suggests that in CF mice there is a vicious circle in which the altered synthesis/secretion of bile salts may reduce the digestion/absorption of cholesterol. As a result, the liver increases the biosynthesis of cholesterol that accumulates in the cells, triggering inflammation and further compromising the metabolism of bile salts.

## Introduction

Cystic fibrosis (CF) is a life-limiting autosomal recessive genetic disorder caused by mutations in the *cystic fibrosis transmembrane conductance regulator* (*CFTR*) gene. These mutations lead to a defective transport of chloride and other ions through the respiratory, biliary, gastrointestinal and reproductive epithelia causing the secretion of a thick mucus [1]. In particular, the mucus obstructs the secretion of pancreatic enzymes in the intestine, giving rise to pancreatic insufficiency (PI), that causes the altered digestion [2, 3] and absorption of lipids [4].

In a previous study [5], we evaluated the cholesterol metabolism in patients with CF by analyzing surrogate biomarkers [6]. In particular, we determined the plasma levels of campesterol and β-sitosterol (phytosterols), which are indirect biomarkers of intestinal cholesterol absorption, and lathosterol, as an indirect marker of the endogenous biosynthesis of cholesterol. The study showed a reduced intestinal absorption of sterols along with an increase in endogenous biosynthesis of cholesterol in patients with CF in comparison with unaffected human subjects. However, despite the increase in liver synthesis, plasma cholesterol levels in patients with CF were lower [5]. This may be due to a partial retention of endogenous cholesterol in hepatocytes, due to a defective cholesterol exocytosis in patients with CF, as previously demonstrated in CF cell lines and animal tissues [7].

Subsequently, we observed that CF patients with PI had a significantly lower absorption of cholesterol and vitamin E than CF patients with pancreatic sufficiency (PS) and healthy controls. This finding suggests that the supplementation of pancreatic enzymes is not sufficient to allow a normal lipid digestion in CF patients with PI [8] probably due to the small amount of cholesterol esterase in supplementation therapy [9] as well as to the reduced synthesis, secretion [10] and reabsorption of bile salts [11] caused by different degrees of CF liver disease (CFLD) [12, 13].

Therefore, the aims of the present study were to investigate cholesterol metabolism in CF mice in comparison with *wild type* (WT) mice and to evaluate the effects of a cholesterol supplementation on cholesterol metabolism in both CF and WT mice. In particular, we evaluated: i) the intestinal absorption of cholesterol, testing serum levels of cholesterol and phytosterols [6]; ii) the liver cholesterol endocytosis, testing the liver gene expression of *LDL receptor* (*LDLR*) [14]; iii) the endogenous cholesterol biosynthesis, evaluating the liver gene expression of *3-hydroxy-3-methylglutaryl-CoA reductase* (*HMG-CoAR*), which encodes for the key enzyme of the synthesis pathway [15, 16], and the levels of liver lathosterol, which is a precursor of the endogenous cholesterol synthesis [6, 16]; iv) the liver cholesterol catabolism, evaluating the liver gene expression of *acyl CoA*:*cholesterol acyl transferase 2* (*ACAT2*), which is involved in the esterification of hepatic cholesterol [17], and *cytochrome P450 7A1* (*CYP7A1*), which is the key enzyme of bile salts synthesis [10, 18]; v) the levels of liver cholestanol as an intermediate product of bile salts synthesis [10, 18]; vi) the liver cholesterol to define the levels of cholesterol accumulation in this organ; and vii) the liver gene expression of *tumor necrosis factor alpha* (*TNFα*), as a marker of inflammation [19].

## Animals and methods

### Mice and treatments

Cystic fibrosis mice homozygous for the F508del *CFTR* mutation in the FVB/129 outbred background (Cftrtm1EUR, F508del, FVB/129) and WT littermates, male and female, were obtained from Bob Scholte, Erasmus Medical Center Rotterdam, The Netherlands: CF coordinated action program EU FP6 LSHMCT- 2005–018932 [20].

The first group of 6 weeks old, CF (n = 3) and WT (n = 3) mice were fed with CF diet (Charles River V1124-70) enriched of linoleic acid and vitamin E for 14 weeks. The second group of 6 weeks old CF (n = 4) and WT (n = 4) mice were fed with the same CF diet for 10 weeks and then they were fed with a diet enriched with cholesterol (1% wt/wt) for 4 weeks (Envigo TD02026). At the end of the last daily treatment, the mice were anesthetized with Avertin (tribromoethanol, 250 mg/kg, Sigma-Aldrich, T48402) and then killed. Blood and liver samples were collected and stored at -80°C [20]. In particular, the blood was collected from retro-orbital sinus by a capillary.

All the procedures in mice were approved by the local Ethics Committee for Animal Welfare of the San Raffaele Scientific Institute of Milan (IACUC No. 849) and were carried out in strict respect of European and National regulations for animal use in research (2010/63 UE).

### Sterol profile analysis

For the analysis of serum sterols, blood samples were collected in plastic tubes without anticoagulant/additive and stored at -80°C immediately after collection. Before sterols extraction, each blood sample was centrifuged at 18220 g for 10 min at 4°C and 100 μL of supernatant (hemolyzed serum) was collected and placed in a pyrex tube. To precipitate the hemoglobin, 1.7 mL of ice cold ethanol was added to the sample, vigorously mixed and incubated in ice for 15 min. The sample was then centrifuged at 3000 rpm for 25 min and the supernatant was transferred in a second pyrex tube for the sterols extraction performed as previously described [5, 21]. Briefly, the sample was mixed with 40 μg of 5α-cholestane (internal standard) and hydrolyzed by incubation in 1N potassium hydroxide ethanolic solution at 80°C for 1 hour. The sterols were then extracted by hexane and derivatized by N,O-bis(trimethylsilyl)trifluoroacetamide (BSTFA) and pyridine, dried under nitrogen, and dissolved in 100 μL of dichloromethane. For qualitative sterols analysis, 1 μL of solution was injected in a gas chromatograph coupled with a mass spectrometer (GC-MS; GC 7890 A/MD 5975C, Agilent Technologies, Santa Clara, CA, USA) in scan mode from m/z 50 to 500. While, quantitative analysis was carried out by injecting 1 μL of solution in a gas chromatograph coupled with a flame ionization detector (GC-FID; HP-5890, Agilent Technologies). Both instrumentations were equipped with Elite-5MS capillary column (PerkinElmer, USA).

For the analysis of sterols in the liver, the tissue samples were first weighed and the homogenates were subsequently prepared as follows. The liver tissue was placed in a clean plastic vial with the butylated hydroxytoluene (1 mg/g of tissue), as an antioxidant [22], and 400–800 μL of distilled water, based on the weight of the tissue, and homogenized using an Ultra Turrax T25 digital homogenizer (IKA®-Werke GmbH & Co. KG, Staufen, Germany). The sterols were extracted from 100 μL of liver homogenate and analyzed as described above for serum. The levels of sterols in liver were normalized by the tissue weight and expressed as μg/mg of tissue for cholesterol and ng/mg of tissue for other sterols.

The analytical solvents of HPLC grade were obtained from Sigma (St Louis, MO, USA). Potassium hydroxide was purchased from Merck (Merck KGaA, Darmstadt, Germany). BSTFA was obtained from Sigma (St Louis, MO, USA). Stock solutions of standard sterols (Sigma, St Louis, MO, USA) were prepared in chloroform/methanol (2:1, v/v) at a concentration of 1 mg/mL. For 5α-cholestane (internal standard for sterol analysis), a work solution at a concentration of 0.4 mg/mL was prepared.

### RNA isolation, qRT-PCR

We analyzed by qRT-PCR the levels of expression of the following genes: *LDLR*, *HMG-CoAR*, *ACAT2*, *CYP7A1* and *TNFα*. All the used primer sequences were listed in S1 Table. The total RNA was extracted from the mouse liver tissue using TriZol (Invitrogen, Waltham, MA, USA) according to the manufacturer’s instructions, and the genomic DNA was removed by treating with DNAse enzyme. The RNA concentration and the purity were measured using NanoDrop 2000 (Thermo Scientific). RNA (1 μg) was converted to cDNA by reverse transcription using iScript cDNA synthesis kit (Bio-Rad).

qRT-PCR was performed using iQ SYBR Green Supermix (Bio-Rad) and the ABI 7900 HT Real Time PCR System (Applied Biosystems). The qRT-PCR results were normalized on expression levels of β-2–microglobulin, used as an internal reference. The results were expressed as relative gene expression levels by using the 2^-ΔCT formula.

### Statistical analysis

Data were reported as mean and standard deviation or mean and standard error where specified. Comparisons between two groups were performed by unpaired Student t test and test type (for equal or unequal variances) was selected by Fisher test. Comparisons among four groups were performed by Kruskal-Wallis and Mann–Whitney U as post hoc test. The significance was accepted at the level of p < 0.05. Statistical analyses were performed with Microsoft Excel software (version 11.0, Microsoft, Redmond, WA, USA) and SPSS software ver. 26.0 (IBM, Armonk, NY, USA).

## Results

### Cholesterol metabolism and the effects of cholesterol supplementation in WT mice

We compared the WT mice with the cholesterol-enriched diet and the WT mice without supplementation. As shown in Table 1, supplemented mice showed significantly lower levels of serum phytosterols (7.6 fold) and liver lathosterol (7.5 fold) and significantly higher levels of liver cholesterol (6.2 fold) and cholestanol (4.2 fold). Serum cholesterol was significantly higher (1.3 fold) in cholesterol-supplemented mice than in mice without supplementation.

**Table 1:** Comparison of sterol levels in WT mice with and without cholesterol-supplementation.

	Serum		Liver		
Mouse populations	Phytosterols (mg/dL)	Cholesterol (mg/dL)	Lathosterol (ng/mg tissue)	Cholesterol (μg/mg tissue)	Cholestanol (ng/mg tissue)
WT with cholesterol supplementation	0.25 (0.08)^a^	122.1 (8.3)^b^	< 0.2^b^^,^^§^	15.1 (4.6)^a^	36.0 (7.7)^b^
WT without supplementation	1.92 (0.37)	89.9 (2.5)	1.5 (0.1)	2.1 (0.2)	8.7 (1.7)

^a^p < 0.05

^b^p < 0.005; unpaired Student t test.

^§^Limit of quantification (LOQ) of the method. WT: *wild type* mice.

**Fig 1:**
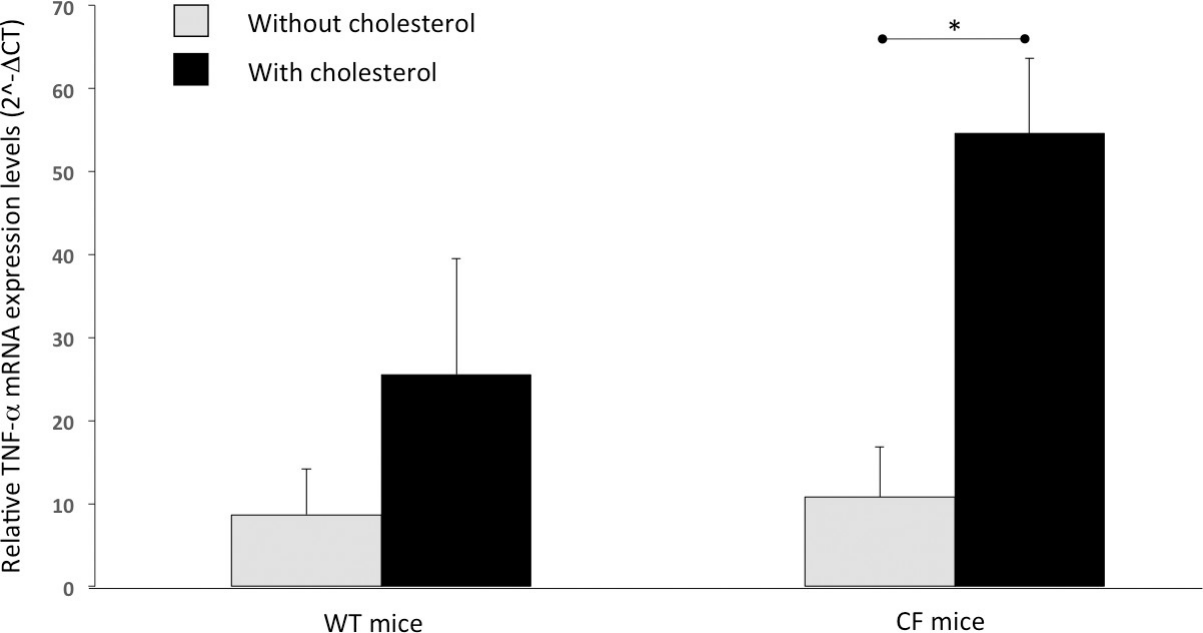
Liver expression of TNFα mRNA normalized on β-2–microglobulin expression levels. Error bars correspond to standard error. Comparisons between two groups were performed by unpaired Student t test. * p < 0.05.

Furthermore, as shown in Table 2, WT mice fed with cholesterol supplementation had significantly lower levels of liver *LDLR* (2.5 fold) and *HMG-CoAR* (2.2 fold) mRNA, while *ACAT2* and *CYP7A1* mRNA levels resulted significantly higher (2.9 and 6.0 fold, respectively). Finally, the liver mRNA levels of *TNFα* showed an increasing trend in cholesterol-supplemented WT mice (Fig 1).

**Table 2:** Comparison of liver mRNA expression in WT mice with and without cholesterol-supplementation.

Mouse populations	*LDLR*	*HMG-CoAR*	*ACAT2*	*CYP7A1*
WT with cholesterol supplementation	1.0 (0.3)^b^	5.3 (1.0)^a^	22.7 (3.7)^b^	11.8 (6.1)^a^
WT without supplementation	2.5 (0.5)	11.7 (3.7)	7.8 (0.8)	2.0 (1.5)

^a^p < 0.05

^b^p < 0.005; unpaired Student t test. *ACAT2*: *acyl CoA*:*cholesterol acyl transferase 2; CYP7A1*: *cytochrome P450 7A1; HMG-CoAR*: *3-hydroxy-3-methylglutaryl-CoA reductase; LDLR*: *LDL receptor;* WT: *wild type* mice.

### Comparison of cholesterol metabolism between CF and WT mice fed without supplementation

We compared the CF mice and the WT mice without supplementation. As shown in Table 3, CF mice had significantly lower levels of serum phytosterols (2.0 fold) together with a decreasing trend of serum cholesterol. On the other hand, liver lathosterol (3.1 fold) and cholesterol (1.2 fold) levels were significantly higher with an increasing trend of liver cholestanol.

**Table 3:** Comparison of sterol levels in CF and WT mice without cholesterol-supplementation.

	Serum		Liver		
Mouse populations	Phytosterols (mg/dL)	Cholesterol (mg/dL)	Lathosterol (ng/mg tissue)	Cholesterol (μg/mg tissue)	Cholestanol (ng/mg tissue)
CF without supplementation	0.97 (0.14)^a^	81.7 (6.3)	4.7 (0.7)^a^	2.6 (0.1)^a^	9.5 (1.1)
WT without supplementation	1.92 (0.37)	89.9 (2.5)	1.5 (0.1)	2.1 (0.2)	8.7 (1.7)

^a^p < 0.05; unpaired Student t test. CF: Cystic fibrosis mice; WT: *wild type* mice.

Furthermore, as shown in Table 4, CF mice had significantly higher *HMG-CoA*R (18.0 fold) and *CYP7A1* (16.3 fold) mRNA levels with an increasing trend of liver *LDLR* and *ACAT2* mRNA levels, in comparison with the WT mice. Finally, liver mRNA levels of *TNFα* showed an increasing trend in CF mice (Fig 1).

**Table 4:** Comparison of liver mRNA expression in CF and WT mice without cholesterol-supplementation.

Mouse populations	*LDLR*	*HMG-CoAR*	*ACAT2*	*CYP7A1*
CF without supplementation	5.7 (2.4)	207.0 (52.1)^a^	18.4 (6.2)	32.7 (4.8)^b^
WT without supplementation	2.5 (0.5)	11.7 (3.7)	7.8 (0.8)	2.0 (1.5)

^a^p < 0.05

^b^p < 0.005; unpaired Student t test. *ACAT2*: *acyl CoA*:*cholesterol acyl transferase 2;* CF: Cystic fibrosis mice; *CYP7A1*: *cytochrome P450 7A1; HMG-CoAR*: *3-hydroxy-3-methylglutaryl-CoA reductase; LDLR*: *LDL receptor;* WT: *wild type* mice.

### The effects of cholesterol supplementation in CF mice

We compared the CF mice with cholesterol supplementation *versus* the WT mice with cholesterol supplementation and the CF mice without supplementation. In comparison with the supplemented WT mice, the supplemented CF mice showed: i) significantly higher levels of serum phytosterols (2.0 folds) and liver lathosterol (22.0 fold); ii) significantly lower levels of both serum (1.3 fold) and liver cholesterol (4.3 fold) together with significantly lower liver cholestanol (3.4 fold) (Table 5).

**Table 5:** Sterol levels in supplemented CF mice in comparison to supplemented WT and no supplemented CF mice.

	Serum		Liver		
Mouse populations	Phytosterols (mg/dL)	Cholesterol (mg/dL)	Lathosterol (ng/mg tissue)	Cholesterol (μg/mg tissue)	Cholestanol (ng/mg tissue)
A) WT with cholesterol supplementation	0.25 (0.08)	122.1 (8.3)	< 0.2	15.1 (4.6)	36.0 (7.7)
p value (A vs B)^a^	< 0.005	< 0.05	< 0.005	< 0.05	< 0.005
B) CF with cholesterol supplementation	0.49 (0.06)	95.9 (9.0)	4.4 (1.2)	3.5 (0.2)	10.7 (3.1)
p value (B vs C)^a^	< 0.005	n.s.	n.s.	< 0.05	n.s.
C) CF without supplementation	0.97 (0.14)	81.7 (6.3)	4.7 (0.7)	2.6 (0.1)	9.5 (1.1)

^a^Unpaired Student t test. n.s.: not significant, p > 0.05. CF: Cystic fibrosis mice; WT: *wild type* mice.

Furthermore, as shown in Table 6, the supplemented CF mice had significantly higher levels of liver *LDLR* (4.7 fold), *HMG-CoAR* (41.2 fold) and *CYP7A1* (5.3 fold) mRNA levels, in comparison with the supplemented WT mice, while the liver mRNA levels of *ACAT2* were not significantly different (Table 6). Liver mRNA levels of *TNFα* were higher in CF mice, although not significant (Fig 1).

**Table 6:** Liver mRNA expression in supplemented CF mice in comparison to supplemented WT and no supplemented CF mice.

Mouse populations	*LDLR*	*HMG-CoAR*	*ACAT2*	*CYP7A1*
A) WT with cholesterol supplementation	1.0 (0.3)	5.3 (1.0)	22.7 (3.7)	11.8 (6.1)
p value (A vs B)^a^	n.s.	< 0.05	n.s.	< 0.05
B) CF with cholesterol supplementation	4.7 (3.0)	206.0 (38.1)	29.3 (18.4)	63.2 (40.0)
p value (B vs C)^a^	n.s.	n.s.	n.s.	n.s.
C) CF without supplementation	5.7 (2.4)	207.0 (52.1)	18.4 (6.2)	32.7 (4.8)

^a^Unpaired Student t test. n.s.: not significant, p > 0.05. *ACAT2*: *acyl CoA*:*cholesterol acyl transferase 2;* CF: Cystic fibrosis mice; *CYP7A1*: *cytochrome P450 7A1; HMG-CoAR*: *3-hydroxy-3-methylglutaryl-CoA reductase; LDLR*: *LDL receptor;* WT: *wild type* mice.

Comparing the CF mice with and without supplementation (Table 5 and 6), we observed that all the parameters resulted not significantly different, except for serum phytosterols levels, that were significantly lower in the supplemented CF mice (2.0 fold), and the liver cholesterol and *TNFα* mRNA levels that were significantly higher in the supplemented CF mice (1.3 fold and 5.0 fold, respectively; Fig 1).

**Fig 2:**
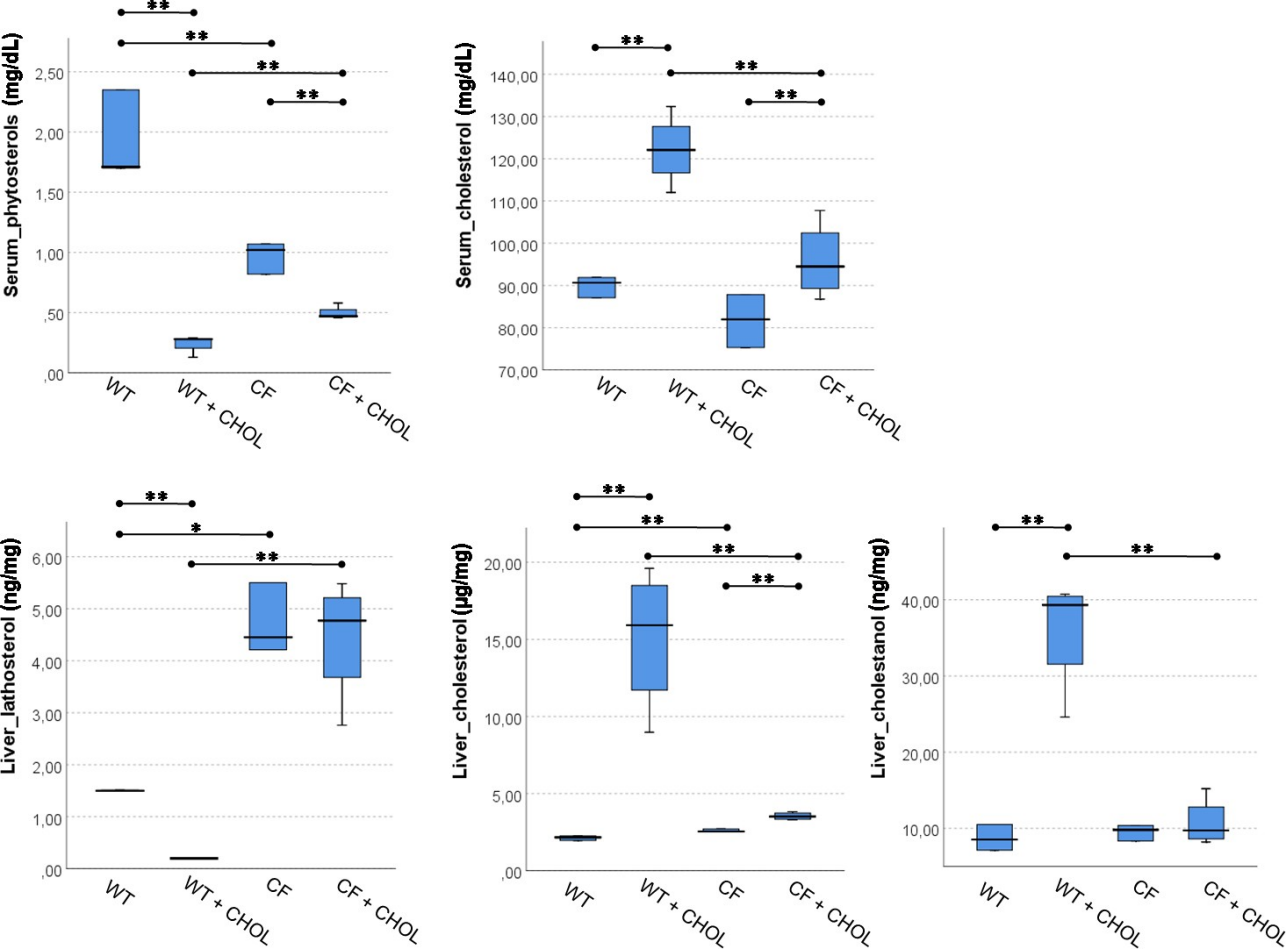
Multiple comparison among WT and CF with and without cholesterol supplementation for serum and liver sterols. The significance of Kruskal-Wallis test for serum phytosterols, liver lathosterol and liver cholesterol was < 0.0001. The significance for serum cholesterol and liver cholestanol was < 0.002. Mann–Whitney U post hoc test: * p < 0.05, ** p < 0.005.

**Fig 3:**
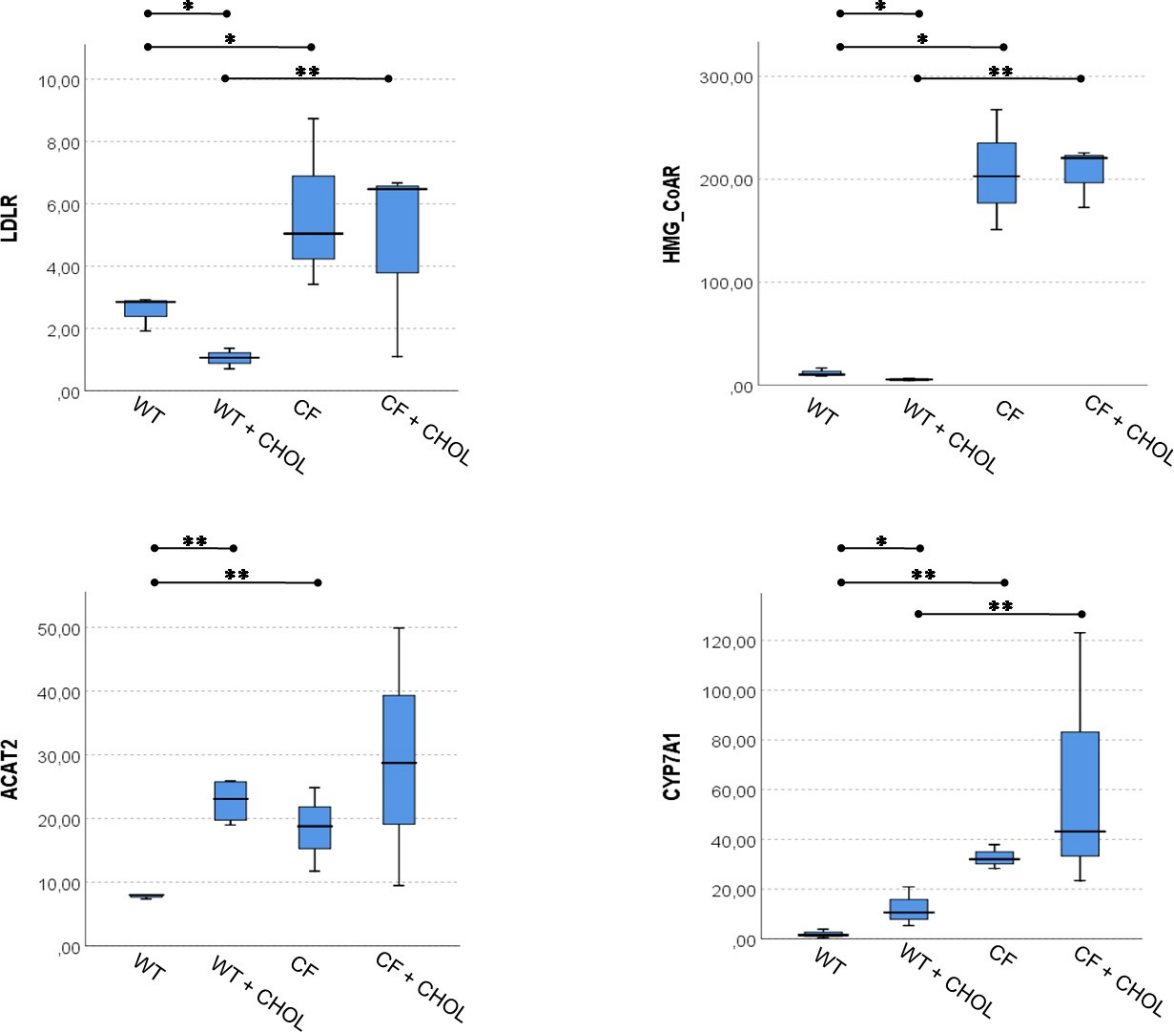
Multiple comparison among WT and CF with and without cholesterol supplementation for liver mRNA expression levels of *LDLR*, *HMG-CoAR*, *ACAT2* and *CYP7A1*. The significance of Kruskal-Wallis test for all variables was < 0.0001. Mann–Whitney U post hoc test: * p < 0.05, ** p < 0.005.

The comparison among all four groups of mice were reported in Fig 2, for serum and liver sterols, and in Fig 3, for liver mRNA levels of *LDLR*, *HMG-CoAR*, *ACAT2* and *CYP7A1*. Overall, these multiple comparisons summarize all the study results, which are reported in detail in Tables 1–6.

## Discussion

Our preliminary study shows that the cholesterol supplementation in WT mice causes a series of biochemical changes similar to those physiologically observed in humans [10, 14–17]. Specifically we observed: i) an increase of cholesterol absorption, in agreement with Schwarz et al. [23]; ii) a reduction of serum phytosterol due to the well-known competition of cholesterol with phytosterols for intestinal absorption [24]; iii) the inhibition of *LDLR* gene expression due to the increased amount of liver cholesterol; iv) the inhibition of endogenous biosynthesis of cholesterol, supported by the reduction of liver gene expression of *HMG-CoAR* and the consequent reduction of liver levels of lathosterol; and v) the activation of liver cholesterol catabolism supported by the enhanced gene expression of *ACAT2*, in agreement with Repa et al. [25], and *CYP7A1*, which induce the esterification of cholesterol and the synthesis of bile salts, respectively, and the increase of liver cholestanol, a catabolic product of acidic pathway of bile salts [18]. We also observed an increasing trend of *TNFα* mRNA levels in agreement with Crescenzo et al. [26] that found a significant increase of plasma TNFα levels in high fat fed rats. Overall these effects, comparable to those observed in humans suggest that the mouse may be a suitable preclinical model to study cholesterol metabolism.

For the first time we showed that, in comparison with WT mice, CF mice display a series of metabolic alterations at baseline (without supplementation) similar to those observed in patients with CF [5, 8]. Specifically we observed: i) a reduced intestinal absorption of sterols, supported by the lower levels of serum phytosterols; ii) an increased endogenous biosynthesis of cholesterol, confirmed by the very high mRNA levels of liver *HMG-CoAR* and by the higher concentrations of liver lathosterol. Interestingly, despite the enhanced synthesis of cholesterol in the CF liver and the higher levels of liver cholesterol, the levels of serum cholesterol in CF mice are lower than in WT. These data suggest that in CF mice there are impaired liver mechanisms of cholesterol secretion, as previously reported in CF cell models and tissues from CF mice [27, 28]. These studies reported an enhanced *de novo* synthesis of cholesterol, followed by its accumulation at endo-lysosomal level due to a block in the translocation to the Golgi and endoplasmic reticulum (ER). They postulated that the accumulation was caused by the misfolded membrane proteins, that escape ER quality control and impact on lipid homeostasis [29]. The lack of cholesterol provision to the ER is followed by the activation of sterol regulatory element-binding protein that enhances the endogenous synthesis of cholesterol [7]. In addition, our findings suggest that, in CF mice liver, there is an enhanced biosynthesis of endogenous cholesterol due to the lack of inhibition of *HMG-CoAR*, as previously observed in cell models [7, 27], that could be due to a lack of unidentified endogenous regulatory factors that cause the accumulation of free cholesterol in the liver. Despite this, we also observed a lack of inhibition of *LDLR* gene expression by the high levels of cholesterol in the CF liver, not previously reported by others in cell models. It seems that such accumulation triggers inflammation, as suggested by the higher levels of *TNFα* gene expression that we observed in CF mice. Interestingly, the liver of CF mice enhances the gene expression of *ACAT2*, encoding for the enzyme that esterifies cholesterol before its secretion [17, 25], but this activation is not followed by a significant release of cholesterol in the blood. Similarly, the liver of CF mice expresses very high mRNA levels of *CYP7A1*, which encodes for the key enzyme of bile salts synthesis [10], although the levels of liver cholestanol, an intermediate of bile salt synthesis [10], are similar to those observed in WT mice suggesting that this pathway may be also impaired.

The cholesterol supplementation in CF mice causes an increase of serum cholesterol (even if less significant of that observed in supplemented WT) and consequently a significant reduction of serum phytosterols (about 50% less) as compared to CF mice with no supplementation, indicating that the supplementation favors some absorption of cholesterol in CF mice. However, the supplementation promotes a further increase (+ 35%) of cholesterol in the liver, in comparison with CF mice without supplementation, which is due to the above mentioned alterations in the CF liver, which further increases the inflammatory reaction, supported by the considerable gene expression of *TNFα*. Cholesterol supplementation has no effect on *LDLR*, *HMG-CoAR*, *ACAT2* and *CYP7A1* expression due to mechanisms to be investigated yet. In particular, the altered mRNA expression levels of *LDLR*, *HMG-CoAR*, *ACAT2* and *CYP7A1* in CF mice suggest that the regulatory system of cholesterol homeostasis is already stimulated at baseline and it is not affected by the cholesterol supplementation, although the response is very heterogeneous.

A limitation of this study is represented by the small number of mice. This depended on the high mortality rate of CF mice [30] and, at the same time, on the aim of studying a CF mice group as homogeneous as possible.

## Conclusions

Our results show that in CF mice there is an impairment of intestinal cholesterol absorption and liver cholesterol metabolism (among which an alteration of *HMG-CoAR* regulation of cholesterol synthesis and an impairment of the mechanism that regulates the *LDLR* expression by the liver). Although these preliminary results should be confirmed by future experiments, it seems that a vicious circle occurs, in which the altered synthesis and secretion of biliary salts contribute to reducing cholesterol digestion and absorption; consequently, there is an enhanced liver biosynthesis of cholesterol that accumulates in the cell triggering inflammation that involves small bile ducts further impairing the synthesis and release of biliary salts.

## Supporting information

S1 Table Primer sequences used for qRT-PCR. (DOC)
