# Supplementary material for: Impaired cholesterol metabolism in the mouse model of cystic fibrosis. A preliminary study
Source: PLoS One. 2021 Jan 7;16(1):e0245302. doi: 10.1371/journal.pone.0245302 (PMC7790534; doi:10.1371/journal.pone.0245302)
Supplement: S1 Table — (DOC) [file pone.0245302.s001.doc]

**S1 Table. Primer sequences used for qRT-PCR.**

| **Gene** | **Forward** | **Reverse** | **Ref.** |
| --- | --- | --- | --- |
| B2M | GGTCTTTCTGGTGCTTGTCT | TATGTTCGGCTTCCCATTCTC | 1 |
| HMG-CoAR | CAGGATGCAGCACAGAATGT | CTTTGCATGCTCCTTGAACA | 2 |
| LDLR | ACCCCTCAAGACAGATGGTC | CAGCCCAGCTTTGCTCTTAT | 2 |
| CYP7A1 | GAGCCCTGAAGCAATGAAAG | GCTGTCCGGATATTCAAGGA | 3 |
| ACAT2 | CATGTTCATAGCGGGCCTA | CGTAGACAGGAACATGGGAA | - |
| TNFα | TCGTAGCAAACCACCAAGTG | CCTTGAAGAGAACCTGGGAGT | 4 |

**References**

1. DiFranco KM, Mulligan JK, Sumal AS, Diamond G. Induction of CFTR gene expression by 1,25(OH)2 vitamin D3, 25OH vitamin D3, and vitamin D3 in cultured human airway epithelial cells and in mouse airways. J Steroid Biochem Mol Biol. 2017;173: 323-32.
2. Wu N, Sarna LK, Hwang SY, Zhu Q, Wang P, Siow YL, O K. Activation of 3-hydroxy-3-methylglutaryl coenzyme A (HMG-CoA) reductase during high fat diet feeding, Biochim Biophys Acta. 2013;1832: 1560-8.
3. Lu Y, Du Y, Qin L, Wu D, Wang W, Ling L, et al. Gypenosides altered hepatic bile acids homeostasis in mice treated with high fat diet. Evid Based Complement Alternat Med. 2018;2018: 8098059.
4. Yang G, Parkhurst CN, Hayes S, Gan WB. Peripheral elevation of TNF-α leads to early synaptic abnormalities in the mouse somatosensory cortex in experimental autoimmune encephalomyelitis. Proc Natl Acad Sci U S A. 2013;110: 10306-11.
